# Accelerated Hypertension after Venlafaxine Usage

**DOI:** 10.1155/2014/659715

**Published:** 2014-09-24

**Authors:** Yüksel Kıvrak, Tolga Sinan Güvenç, Nurcihan Akbulut, İbrahim Yağcı, Gülşen Çığşar, Süleyman Gündüz, Bahattin Balcı

**Affiliations:** ^1^Department of Psychiatry, Kafkas University School of Medicine, 36000 Kars, Turkey; ^2^Department of Cardiology, Kafkas University School of Medicine, Kars, Turkey; ^3^Department of Emergency, Kafkas University School of Medicine, Kars, Turkey; ^4^Department of Psychiatry, Kars State Hospital, Kars, Turkey

## Abstract

Venlafaxine is the first antidepressant that acts via inhibiting serotonin and noradrenaline reuptake. Hypertension is observed in doses exceeding 300 mg/day and is the most feared complication. We report a patient with accelerated hypertension after venlafaxine use observed at a dose of 150 mg/day. A 23-year-old patient with symptoms of insomnia, depression, anhedonia, fatigue admitted our clinic. Venlafaxine at a dose of 75 mg/day was initiated after he was diagnosed with major depressive disorder. After 5 months, venlafaxine dose was uptitrated to 150 mg/day due to inadequate response to drug. After using venlafaxine for ten months at the dose of 150 mg/day, he admitted our clinic with headache and epistaxis. He was hospitalized after his blood pressure was measured as 210/170 mmHg. No secondary causes for hypertension were found, and venlafaxine treatment was considered possible etiologic factor. After stopping venlafaxine treatment, his blood pressure was reverted back to normal limits. While mild elevation of blood pressure could be observed after venlafaxine treatment, this case shows that accelerated hypertension with a diastolic blood pressure rise above 120 mmHg could be observed at relatively low doses of venlafaxine. Close monitoring of blood pressure is necessary after initiation of treatment, as accelerated hypertension could cause endorgan damage with potentially catastrophic results.

## 1. Introduction

Major depressive disorder (MDD) is the fourth most common disease diagnosed worldwide, with an annual prevalence of 6.6% and a lifelong prevalence of 16.2% [1]. It affects both genders and is prevalent in all age groups.

Venlafaxine is the first product that was commercially available for the treatment of major depression among all the serotonin-norepinephrine reuptake inhibitors [2]. At high doses, the effect of venlafaxine appears earlier than that of the other antidepressants. Adverse effects of venlafaxine include nausea, somnolence, dry mouth, dizziness, nervousness, constipation, asthenia, blurred vision, abnormal ejaculation or orgasm, erectile dysfunction and impotence [2]. However, increase in blood pressure is the most feared complication of venlafaxine, which is usually observed in administration of the doses higher than 300 mg/dL [3]. In most cases, the rise in blood pressure is mild and self-limiting. Accelerated hypertension, in which the diastolic blood pressure rises above 120 mmHg accompanied by complications or alone, is rarely observed with the therapeutic doses and has not been reported before with the use of venlafaxine alone.

In this paper, we report a patient with MDD who experienced accelerated hypertension with blood pressure measurements as high as 210/170 during venlafaxine use. No other secondary causes for hypertension were found, and blood pressure decreased to normal levels right after the drug was stopped.

## 2. Case Report

A 23-year-old male patient diagnosed with major depressive disorder, visited our institution, and was complaining from persistent symptoms despite the antidepressant therapy. At his initial visit, the patient had been suffering from insomnia, depression, anhedonia, irritability, inability to concentrate, and fatigue for about a year.

Psychological examination revealed slowing of movements, reduced facial expressions, and decrease in his self-care. He had normal orientation of time, space, and person.

Cardiovascular examination was normal at the initial visit, with a blood pressure of 120/70 mmHg and a heart rate of 76 beats/per minute. He had no family history for essential hypertension, chronic renal disorders, or similar chronic diseases.

He was diagnosed with major depressive disorder according to the Structured Clinical Interview for DSM-IV (SCID-1) [4] and 75 mg/day venlafaxine was prescribed. The efficacy of the treatment was planned to be evaluated at monthly intervals. However, the patient did not have regular visits and could only be examined five months later.

Despite missing his visits, the patient said that he was strictly obeying the medication schedule. However, his symptoms had not regressed and the dose of venlafaxine was increased to 150 mg/day.

At the 10th month of the therapy, he readmitted our institution with a complaint of headache and epistaxis. On physical examination, his blood pressure was measured as 210/170 mmHg, and he was subsequently hospitalized with a diagnosis of accelerated hypertension.

After hospitalization, his blood pressure was normalised with the administration of a short acting oral ACE inhibitor. Biochemical analyses including urinalysis and renal function tests, echocardiographic examination, and fundoscopic examination were performed to rule out end organ damage and malignant hypertension. Left ventricular dimensions, wall thickness, and ejection fraction were found to be normal on the echocardiography. Both fundoscopy and renal function tests revealed normal results.

Venlafaxine treatment was stopped as it was thought to be the main predisposal factor for the patient's high blood pressure levels, while a secondary cause was continued to be searched. Renal Doppler ultrasound examination revealed normal renal dimensions and parenchyma without any stenosis.

The protein and vinyl mandelic acid levels were measured within normal limits in the 24-hour urine test. Other biochemical analyses, including blood sodium and potassium levels, were all normal. After stopping venlafaxine treatment, a decrease below 140/90 mmHg in the blood pressure was observed; no additional drug use was needed for lowering the blood pressure.

The patient was discharged from hospital one week after the initial admission. A 24-hour ambulatory blood pressure monitoring was performed one month after the index event and average blood pressure values which were measured 2 times a day (in the morning and at night) were below 130/80 mmHg.

## 3. Discussion

Venlafaxine is used in the treatment of major depressive disorder, generalized anxiety disorder, treatment-resistant depression, and chronic pain syndrome [2]. The drug has dual antidepressive effect since it activates both the serotonin and the norepinephrine receptors. Although venlafaxine binds to both the serotonin and the norepinephrine receptors, at therapeutic doses, its affinity to serotonin receptors is 30 times higher than norepinephrine receptors [5, 6].

At higher doses, the drug also inhibits the dopamine reuptake [7]. At doses exceeding 150 mg/day, the adrenergic effects of venlafaxine became more prevalent [8]. While the adverse effect profile of venlafaxine is similar to other antidepressants, it has fewer drug-drug interactions [9].

At lower doses, the adverse effects of venlafaxine, including nausea, vomiting, gastrointestinal adverse effects, and sexual dysfunction, are similar to that of other SSRIs, while at higher doses it becomes similar to that of norepinephrine reuptake inhibitors [6]. While adrenergic effects of venlafaxine appear with doses administrated more than 150 mg/day, apparent increase in blood pressure and increased heart rate are observed when the daily dose exceeds 300 mg [10]. In a previous study by Mbaya et al., 12.5% of the patients who were receiving venlafaxine treatment developed hypertension [11]. According to a review published in 1995, increased blood pressure is seen to be 5% in patients using doses lower than 100 mg/daily, 6% using between 100 and 200 mg daily, and 13% using more than 300 mg daily. It is reported that the use of 300/375 mg day for six weeks causes about 7 mmHg increase in blood pressure [12]. Although, higher doses of venlafaxine are associated with higher hypertension rates, doses below 100 mg/day can also cause hypertension. Polymorphism of the genes CYP2D6 and CYP2C19 which are involved in the metabolism of venlafaxine may change the pharmacokinetics of the agent and cause the adverse side effects at different drug levels. A poor metabolisation activity of the CYP2D6 (maybe in combination with the poor metabolisation activity of CYP2C19) might be the reason for the increased toxicity of the comparatively low dose of venlafaxine [13–16].

In our case, the blood pressure increased after increasing daily venlafaxine dose to 150 mgs. However, the diastolic blood pressure over 120 mmHg was much more than the expected level. Although we did not observe any complications—such as acute encephalopathy, heart failure, or papilledema—in our patient, such an acute and dramatic increase of blood pressure could be life-threatening.

Depressive mood correlates with blood pressure [17, 18] and depression is a risk factor for developing hypertension [19]. Having chronic hypertension is not a risk factor for aggravated blood pressure response during venlafaxine use [20].

To our knowledge, only one case of accelerated hypertension (where diastolic blood pressure raised above 120 mmHg) after venlafaxine use has been reported previously. It was a female patient treated for anxiety, depression, and alcohol abuse, and her blood pressure was measured as 220/140 mmHg nine days after the initiation of risperidone and 75 mg/day venlafaxine [21]. In contrast to our case, this patient was also on risperidone treatment, which could also be responsible for the increase of blood pressure.

Two other cases were reported to have developed hypertension after venlafaxine use; however, in both cases, the diastolic pressure was below 120 mmHg. In the first case, a female patient with high-normal blood pressure became hypertensive one week after the initiation of 150 mg/day venlafaxine treatment. Repeated measurement was planned one week after the first measurement which revealed a blood pressure of 162/110 mmHg [22]. In the second case reported by Eren et al., a previously normotensive elder male patient with panic disorder became hypertensive after six-day use of venlafaxine 225 mg/day along with hydroxyzine 22.5 mg/day and clonazepam 4 mg/day. His blood pressure was measured as 175/95 mmHg two weeks after the venlafaxine administration [23].

Our patient was younger than these patients reported previously, thus we looked for a secondary cause for hypertension. Particularly, pheochromocytoma was searched, as we hypothesised that venlafaxine could enhance hypertensive crises observed in this condition. However, the urine analysis for vanillylmandelic acid, a metabolite for catecholamines, revealed normal results. Urinalysis, renal ultrasound, and renal artery Doppler examinations were all normal, which ruled out the possibility for any renal diseases. Similarly, congenital vascular lesions that may cause hypertension, such as aortic coarctation, were not observed. The dramatic decrease soon after the venlafaxine treatment stopped also pointed out to drug-induced hypertension.

In conclusion, venlafaxine use was identified as the predisposal factor for the accelerated hypertension in our patient. So far, reports and studies showed venlafaxine may cause mild elevation in blood pressure, which are more important in the long term. However, our study suggests that accelerated hypertension with a diastolic blood pressure rise above 120 mmHg could be observed soon after the venlafaxine administration. While hypertensive complications did not develop in our patient, such a sudden increase in blood pressure could cause stroke or similar disabling conditions. Therefore, patients on venlafaxine should be warned for a potential rise in blood pressure, and a regular followup should be planned for hypertension.
